# Sunitinib Treatment of VHL C162F Cells Slows Down Proliferation and Healing Ability via Downregulation of ZHX2 and Confers a Mesenchymal Phenotype

**DOI:** 10.3390/cancers16010034

**Published:** 2023-12-20

**Authors:** Stéphanie Buart, M’boyba Khadija Diop, Isabelle Damei, Salem Chouaib

**Affiliations:** 1 INSERM UMR 1186, Integrative Tumor Immunology and Immunotherapy, Gustave Roussy, Faculty of Medicine, University Paris-Saclay, 94805 Villejuif, France; isabelle.damei@pasteur.fr; 2 Bioinformatics Core Facility, University of Paris-Saclay, 94805 Villejuif, France; mboyba.diop@gustaveroussy.fr; 3 Thumbay Research Institute for Precision Medicine, Gulf Medical University, Ajman 4184, United Arab Emirates

**Keywords:** ccRCCs, VHL-C162F mutation, ZHX2, sunitinib, hypoxia-related genes, epithelial mesenchymal transition

## Abstract

**Simple Summary:**

Mutations of the *von Hippel–Lindau* (*VHL*) gene lead to VHL disease in patients. It is characterized by numerous benign and malignant tumors in different organs, as highly vascularized clear cell renal cell carcinomas (ccRCCs). The aim of this study was to examine the consequences of VHL-C162F mutation on morphology, proliferation, ability to form colony and healing ability of renal carcinoma cells. We found that VHL-C162F cells display a higher healing ability than wild type (WT) VHL cells. This was associated with a high expression of ZHX2, an oncogenic driver of ccRCCs. More importantly, sunitinib treatment resulted in a decreased proliferation of VHL-C162F cells. This was associated with ZHX2 inhibition and downregulation of P-ERK. Such treatment also confers a more mesenchymal profile which is not in favor of survival for patients with VHL-C162F disease.

**Abstract:**

von Hippel-Lindau (VHL) disease, due to mutations of the tumor suppressor *VHL* gene, is a rare hereditary syndrome with a high risk of developing clear cell renal cell carcinoma (ccRCC). We asked whether the VHL-C162F mutation interferes with proliferation, migration, healing and forming colony ability by using wild-type *VHL* (WT VHL) and VHL-C162F reconstituted cells. We then analyzed the in vitro impact of the sunitinib treatment on VHL-C162F cells. We showed that VHL-C162F mutations have no impact on cell morphology, colony formation and migration ability but confer a significant higher healing ability than in WT VHL cells. RNA sequencing analysis revealed that VHL-C162F mutation upregulates genes involved in hypoxia and epithelial mesenchymal transition (EMT) pathways by comparison with VHL WT cells. We next showed a decrease in healing ability in VHL-C162F cells depleting on ZHX2, an oncogenic driver of ccRCC, highlighting the potential involvement of ZHX2 in aggressiveness of the VHL-C162F cells. Moreover, we found that sunitinib treatment inhibits ZHX2 expression and induces a reduced proliferation correlating with downregulation of P-ERK. Sunitinib treatment also conferred a more mesenchymal profile to VHL-C162F cells with significant downregulation of E-cadherin and upregulation of N-cadherin, Slug and AXL. Sunitinib therapy may therefore promote disease progression in VHL-C162F patients.

## 1. Introduction

The VHL disease is characterized by tumors arising in multiple organs [1]. Two types of VHL disease have been reported based on the risk to develop a tumor of the adrenal gland or pheochromocytoma: type 1 has low risk whereas type 2 is associated with a high risk [2,3]. *VHL* mutations have been identified as one of the genetic determinants driving ccRCC development, intratumoral heterogeneity and invasion [4]. Very recently, it has been elegantly shown that patients with VHL disease are at risk of developing spatially and temporally multiple ccRCCs, which offers a valuable opportunity to analyze inter- and intra-tumor heterogeneity of genetic and immune profiles within the same patient [5]. The VHL-C162F mutation, a substitution of a cysteine in phenylalanine in position 162, is classified as type 1 with low risk to develop a pheochromocytoma [6]. Type 1 is characterized by ccRCC and hemangioblastomas, and is associated with disruptive mutations and gross deletions in VHL [7]. The severity of VHL disease associated with VHL-C162F mutation is one of the most severe [6]. Mutations in the *VHL* gene like VHL-C162F mutation cause disruption in HIF degradation, inducing a pseudo-hypoxia state in normoxia. Also, it can induce over-expression of proteins, including vascular endothelial growth factor (VEGF), platelet derived growth factor (PDGF) and transforming growth factor (TGF) and, consequently, an excessive angiogenesis [8]. VHL inactivation or VHL mutations lead to the constitutive stabilization and accumulation of HIF1/2α. This generates a pseudo-hypoxic state leading to a reprogramming and shaping of an unfavorable tumor microenvironment [9] and contributing to ccRCC development. Currently, our knowledge on the relationship between VHL mutations and the outcome of immunotherapy trials is not well established [10,11].

Several studies have indicated that ZHX2, a member of the ZHX (Zinc fingers and homeoboxes) family, has been involved in many types of cancer [12]. It has been reported that this transcription factor interferes with VHL regulation and evidence has been provided that shows it is associated with ccRCC initiation [13,14]. It has been demonstrated that the *VHL* loss of tumor suppressor gene *VHL* in ccRCC resulted in the accumulation of ZHX2 protein in the nucleus and the subsequent decrease in patient survival rate [13,15]. More importantly, Li et al. identifies ZHX2 acting as an oncogenic driver in ccRCC where hypoxia-inducible factors (HIF1 and HIF2) are deregulated [16]. ZHX2 drives proliferation, migration, colony formation and invasion via activating multiple proteins including Twist, Snail, VEGF, MEK, ERK 1/2 [12,17]. Based on these findings, ZHX2 has been identified as a potential new therapeutic target for ccRCC patients [13]. As it has been shown that several ccRCC cell lines resist to a specific HIF2α inhibitor, it is conceivable that ZHX2 could be considered as an innovative target [13].

A characteristic of ccRCC is its high vascularity due to the alterations of the *VHL* gene. Accumulation of HIFs mediates the overexpression of VEGF [18]. Therapeutical approaches targeting HIF such as VEGF inhibitors are the first-line treatments for ccRCC, but the clinical outcome was disappointing given the drug resistance issue [19,20,21]. Sunitinib is a tyrosine kinase inhibitor that has multiple targets as VEGFR [22,23]. Used as standard first-line therapy, it was approved for treatment of metastatic renal cell carcinoma given its potential role in delaying the time of tumor progression and prolonging survival [24,25,26].

In this study, we attempted to elucidate the consequences of the VHL-C162F mutation in shaping RCC functional features. We demonstrated that VHL-C162F mutation increases cell motility. This correlated with an overexpression of ZHX2 and a transcriptional regulation showing an enrichment of hallmarks of hypoxia and epithelial mesenchymal transition in VHL-C162F cells. We also demonstrated that sunitinib treatment impacts VHL-C162F cell phenotype with an upregulation of EMT-related mesenchymal and proliferation associated markers.

## 2. Materials and Methods

### 2.1. Mutated VHL Cell Lines

The 786-0 cell line was isolated from a VHL−/− human ccRCC. The three different VHL mutated cell lines used in this study were generated in the laboratory of S. Richard (Gustave Roussy, Villejuif, France) and were already described [6]. Briefly, 786-0 cells were transfected with a vector allowing the doxycycline-inducible expression of the full-length wild-type sequence *VHL* (786-0 WT VHL, VHL+/+ or WT VHL cells) or the sequence encoding the C162F mutant (786-0 C162F, VHL mutated or VHL-C162F cells). Control cells were generated by the transfection of the empty vector (786-0 EV, VHL−/− or EV cells). These cell lines were genotyped by direct sequencing of *VHL*. The three cell lines were maintained in cultures as described previously [11].

### 2.2. Gene Silencing for ZHX2

Small interfering RNA (siRNAs) transfection was performed with a mix of two pre-designed siRNAs for ZHX2 (FlexiTube siRNA, cat #SI00767977 and FlexiTube siRNA, cat #SI04201414) or with a non-silencing siRNA used as control (NSi; AllStars Negative Control siRNA, cat #SI03650318) purchased from Qiagen, Courtaboeuf, France. Cells were transfected using lipofectamine RNAiMAX transfection reagent (cat #13778-150, Invitrogen, Life Technologies Corp, San Francisco, CA, USA). After 48 h incubation, cells were lysed for analysis.

The efficacity of gene silencing of ZHX2 was first evaluated by RT-qPCR (primers sequences on request), then by western blot. As the mix of 2 siRNA ZHX2 yielded at least 80% target gene knockdown by RT-qPCR in EV and VHL-C162F cells, it was considered to be efficient.

### 2.3. Sunitinib Treatment

Sunitinib treatment was performed with a capsule of sutent (Pfizer, France) provided by Gustave Roussy Pharmacy containing 25 mg of sunitinib. Sunitinib was diluted in nuclease-free water to get a solution at 5 mM final and preserved at 4 °C for maximum 60 days protected from light.

Cells (1 × 10^4^) were seeded in a 96-well plate with 100 µL medium in triplicate, then treated with different doses of sunitinib (0, 0.5, 1, 2, 3, 5 and 7 μM) for 96 h. The supernatants were removed. MTT solution (100 µL) (1 mg/mL, MTT, Sigma-Aldrich St Louis, MO, USA) was added to each well and incubated for 3 h, and then developed with 100 µL of a solution of isopropanol HCl/well for 10 min. The absorbance (OD) was detected at the wavelength of 570 nm with a microplate auto reader (ThermoFisher Scientific Inc., Waltham, MA, USA). The optimal final concentration of sunitinib to use for the in vitro experiment was 2.5 μM.

For western blot analysis, sunitinib treatment was performed for 15 days. The medium was renewed twice a week.

### 2.4. Single Cell Culture by Cell Sorting

Single cell culture in 96-well microplates was performed with the BD *Influx™* Cell Sorter (Becton Dickinson, Franklin Lakes, NJ, USA) which consists in deposing 1 cell by well in 150 μL final complete DMEM medium (Gibco, Thermo Fisher Scientific, Inc., Waltham, MA, USA), supplemented with 5% tetracycline-free fetal calf serum (FCS) (PAA Laboratories Inc, Ontario, Canada), 1% sodium pyruvate, 1% penicillin/streptomycin, 0.2 mg/mL zeocin, 3 µg/mL blasticidin, and 1 µg/mL doxycycline. After sorting, cell culture was grown at 37 °C, 5% CO_2_ for 12 days. Cells were then washed in PBS (Gibco, Thermo Fisher Scientific, Inc., Waltham, MA, USA), fixed and stained during 30 min in a solution containing 25% methanol and 0.01% crystal violet (Sigma-Aldrich St Louis, MO, USA). Crystal violet binds to DNA in the nuclei of mammalian cells, staining them a deep purple and helping to visualize colonies. Upon removal of the fix/stain solution, cells were rinsed carefully with distilled water. The colonies were allowed to dry at room temperature. Stained colonies were counted in each well.

### 2.5. Clonogenic Assay

Clonogenic assays were performed as described previously [11]. 

### 2.6. Transwell Cell Migration Assays

Cells migration assays were performed by seeding 3 × 10^4^ cells of the three cell lines in 100 μl of 0% FCS medium on top of transwell cell culture inserts (24-well inserts, 8 µm pore size, Corning, NY, USA) according to the manufacturer’s instructions. The lower chamber was filled with 700 µL 5% FCS medium as chemoattractants. After incubation for 24 h, the non-migrating cells on top were scraped off and the membranes were fixed for 10 min in 70% ethanol solution and stained for 5–10 min with a solution of 0.2% crystal violet (Sigma-Aldrich St Louis, MO, USA). Cells that had migrated to the underside of the membranes were counted under an inverted microscope. Photographs of the transwell cell culture inserts for each cell line were taken with microscope EVOS cell Imaging Systems (ThermoFisher Scientific Inc., Waltham, MA, USA). Migrating cells were counted.

### 2.7. Wound Healing Assay

Cells were seeded to reach ~80% confluence as a monolayer after 24 h of growth. The cell monolayer was scratched using a pipette tip across the center. After washing with PBS (Gibco, Thermo Fisher Scientific, Inc., Waltham, MA, USA), cells were incubated at 37 °C and 5% CO_2_. During the incubation, photographs of the cell monolayer were taken at 0, 24, 48, 72 and 96 h post-scratch with microscope EVOS cell Imaging Systems (ThermoFisher Scientific Inc., Waltham, MA, USA). The unoccupied areas were measured at the indicated times.

The same protocol was applied to wound healing assay on cell lines transfected with a mix of siRNA ZHX2 purchased from Qiagen, Courtaboeuf, France or on cell lines treated with sunitinib (Pfizer, France). Transfection with siRNA was performed at day 0, just before scratching. Sunitinib was added at 2.5 μM final in the medium at day 0, just after scratching.

### 2.8. RNA-Sequencing and Gene Set Enrichment Analysis

RNA-sequencing and Gene Set Enrichment Analysis was performed as described previously [11]. GSEA was performed with the GSEA platform of the Broad Institute, following guidelines (http://www.broadinstitute.org/gsea/index.jsp (accessed on 18 November 2019).

### 2.9. Western Blots

Western Blots were performed as described previously [11] with the following primary antibodies listed in Table S1. The detection was performed following incubation with horseradish peroxidase (HRP)-conjugated secondary Abs using an enhanced chemiluminescence kit (GE Healthcare, Chicago, IL, USA). Blot images were captured using a ChemiDoc Imaging System (Biorad, Hercules, CA, USA). Western blot quantifications were performed using ImageJ densitometry software (ImageJ 1.54f version, NIH, Bethesda, MD, USA).

### 2.10. KI67 Proliferation

Proliferation analysis of the three cell lines were performed by direct immuno-staining with a fluorochrome-conjugated antibody anti-human Ki67-PerCPefluor710 (clone 20Raj1) (Cat# 46-5699-41, Thermo Fisher Scientific, Inc., Waltham, MA, USA).

Briefly, 0.2 × 10^6^ cells were fixed and permeabilized 30 min at 4 °C, then stained 30 min at room temperature with fluorochrome-conjugated monoclonal Ab anti human Ki67 dilute at 1/50. Stained cells were analyzed by flow cytometry using a FACS Cytoflex flow cytometer (Beckman Coulter Life Sciences, Villepinte, France). Data were processed using FlowJo V10 software (Becton, Dickinson, Ashland, OR, USA).

### 2.11. Data Analysis and Statistics

Graphics and statistical analyses were performed with GraphPad Prism software version 9.00 (GraphPad Software, La Jolla, CA, USA). Values were expressed as means ± SEM. The number of samples (n), representing the number of independent biological replicates, is indicated in the figure legends. The experiments were repeated at least three times per biological replicate and averaged. We used non-parametric statistical tests when the normality could not be assumed or tested (n too small). In this case, statistical comparisons between groups were performed using unpaired two-tailed Mann-Whitney U tests. Otherwise, statistics were analysed using One Way ANOVA followed by Bonferroni or Tukey post-test as indicated in the figure legends. In all cases, *p* < 0.05 was considered statistically significant.

## 3. Results

### 3.1. Cell Morphology, Colony Formation, Migration Abilities and Healing of VHL-C162F Cells as Compared to WT VHL Cells

**Figure 1:**
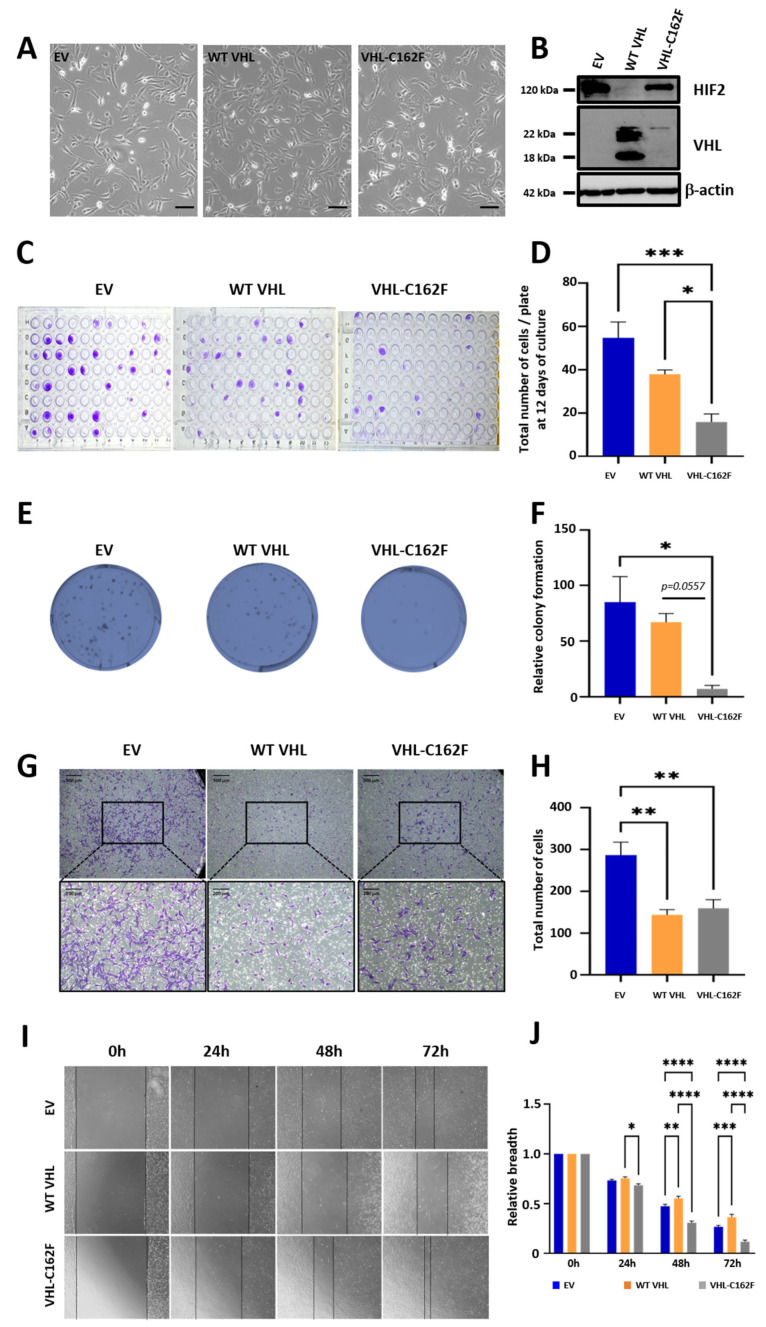
Morphology, colony formation, migration potential and healing ability of C162F VHL mutated cells. (**A**): Phase contrast microscopy images showing the similar morphology of the three cells lines, EV, WT VHL, and VHL-C162F. Scale bar, 100 µm. (**B**): Western blot analysis of HIF-2α and VHL proteins from EV, WT VHL and VHL-C162F cells. β-Actin is used as a loading control. (**C**): Single cell culture by sorting for the three cell lines. Photographs correspond to one representative 96-well microplate. (**D**): The count of the colony corresponds to the total number of EV, WT VHL and C162F cells in one 96-well microplates (*n* = 6). * *p* ≤ 0.05 and *** *p* ≤ 0.001 using One Way ANOVA analysis and Bonferroni test. (**E**): Clonogenic assay using EV, WT VHL, and VHL-C162F cell lines. Photographs correspond to one representative well of a 6-well microplate. (**F**): The count of the colony is the mean of nine wells (*n* = 3). * *p* ≤ 0.05 using One Way ANOVA analysis and Tukey test. (**G**): The migration ability of EV, WT VHL and VHL-C162F was determined by transwell cell migration assays. Respectively, scale bars are 500 μm and 200 μm for photographs up and down. (**H**): The total number of migrated cells is shown in the bar graphs (*n* = 3). ** *p* ≤ 0.01 using One Way ANOVA analysis and Tukey test. (**I**): Representative scratch wound images showing the healing ability in EV, WT VHL and VHL-C162F cells at 0, 24, 48 and 72 h. (**J**): Graphs represent normalized relative breadth measured at 24, 48 and 72 h for the 3 cells lines (*n* = 12). * *p* ≤ 0.05, ** *p* ≤ 0.01, *** *p* ≤ 0.001 and **** *p* ≤ 0.0001 using One Way ANOVA analysis and Bonferroni test. The uncropped bolts are shown in Supplementary Materials Figure S1.

It is known that mutations of *VHL* resulted in a change of cell morphology in ccRCCs [27]. We attempted to analyze the consequences of the VHL-C162F mutation in ccRCCs using the clear-cell renal carcinoma 786-0 cells (VHL−/−) transfected with either empty (EV), WT VHL, or VHL-C162F encoding vectors. We showed that compared to EV cells used as control, cells transfected with WT VHL or VHL-C162F have similar morphology (Figure 1A).

It is also admitted that VHL loss of function due to *VHL* mutations results in HIF accumulation, leading to a pseudo-hypoxic state despite normal oxygen conditions. This pseudo-hypoxic state was observed in protein extracts of VHL-C162F cells by western blot analysis (Figure 1B and Figure S1) showing an expression of HIF-2 associated with low expression of VHL.

We also asked whether VHL-C162F mutation interferes with the proliferative potential in conditions of a single cell culture by sorting (Figure 1C) and to form colonies at low dilution (Figure 1E). We showed that the 786-0 cells transfected with C162F-VHL had a reduced potential to grow as a single cell and as colonies, compared with the WT VHL-and EV-transfected 786-0 cells (Figure 1D,F).

In addition, we tested the ability of migration via transwell assay (Figure 1G). We found no significant difference between WT VHL and VHL-C162F cells (Figure 1H). We asked whether if VHL-C162F mutation can impact healing abilities by a scratch test of wound healing assay at high confluence (Figure 1I). We obtained data indicating that VHL-C162F transfected cells had an increased healing ability as compared to WT VHL and EV cells (Figure 1J). We concluded that VHL-C162F cells are less aggressive at low cell density.

### 3.2. Comparative Analysis of Transcriptional Profile of WT VHL and VHL-C162F Showed Less Variation than between EV and VHL-C162F

**Figure 2:**
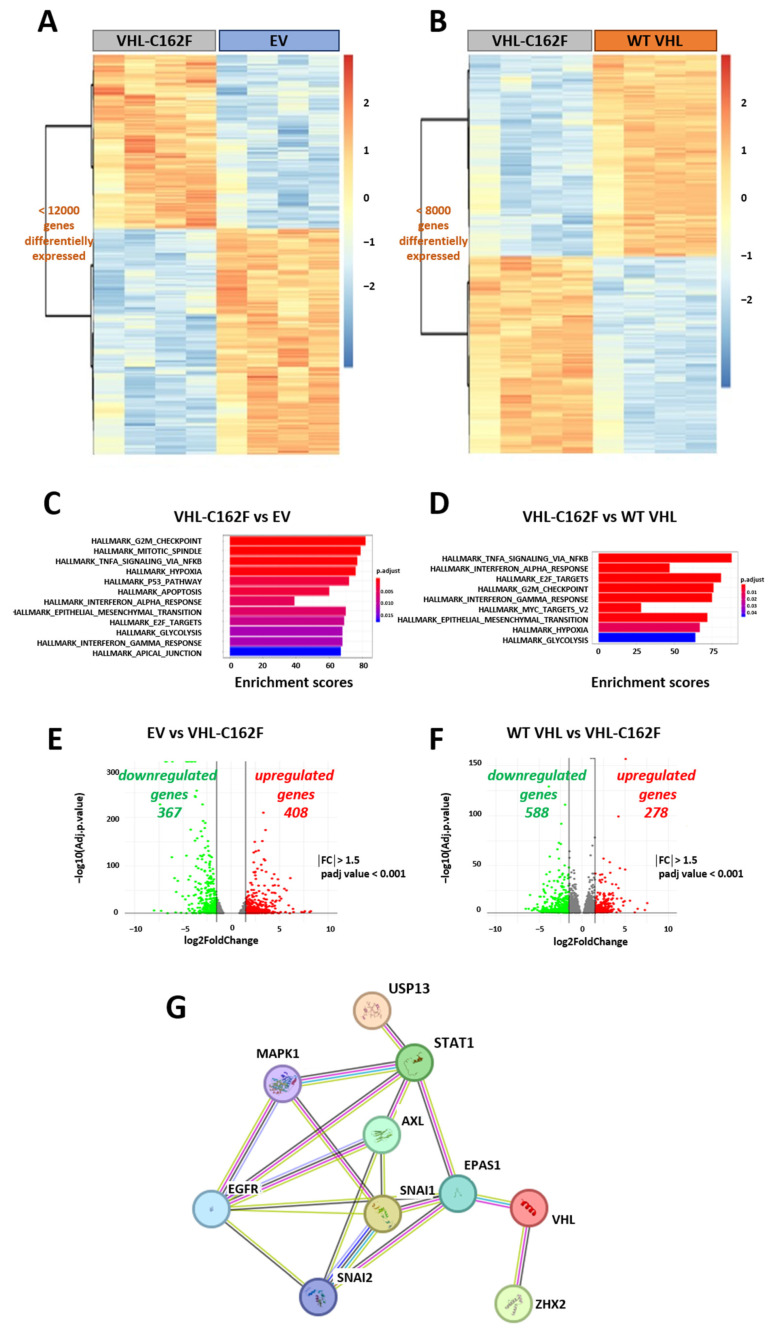
RNA-Seq analysis revealed discrete transcriptional differences between WT VHL and VHL-C162F cells reflecting an increase in cell plasticity in mutated cells. (**A**): Heatmap from RNA-Seq analysis comparing EV and VHL-C162F cells in triplicate. (**B**): Heatmap from RNA-Seq analysis comparing WT VHL and VHL-C162F cells in triplicate. (**C**): Differentially enriched gene sets nominated from GSEA analysis in VHL-C162F vs. EV cells classified by enrichment scores. A positive score indicates enrichment. (**D**): Differentially enriched gene sets nominated from GSEA analysis in VHL-C162F vs. WT VHL cells classified by enrichment scores. A positive score indicates enrichment. (**E**): Volcano plot of genes differentially expressed (Log10 fold change) in VHL-C162F vs. EV cells and (**F**): in VHL-C162F cells vs. WT VHL cells. For VHL-C162F vs. EV, a total of 367 downregulated genes and 408 upregulated genes were identified with a fold change >1.5, FDR < 0.001. For VHL-C162F vs. WT VHL, a total of 588 downregulated genes and 278 upregulated genes were identified with a fold change >1.5, padj value < 0.001. (**G**): Protein–Protein Interaction Networks showing Functional Enrichment Analysis by STRING. VHL interacts with some proteins as EPAS1 (HIF2α), STAT1, USP13, ZHX2, EGFR, AXL, SNAI1, SNAI2 (Slug) and MAPK1.

We next compared the transcriptomic profiles of transfected 786-0 cells EV, WT VHL, and C162F-VHL using RNA-Seq (Figure 2).

Data shown in Figure 2 indicate distinct transcriptomic profiles between EV and VHL-C162F transfected cells (Figure 2A) and between WT VHL and VHL-C162F cells (Figure 2B). Results depicted in Figure 2C,D illustrate two comparative analyses performed to examine the transcriptomic profiles changes in EV, VHL-C162F and in WT VHL cells. Volcano plot analysis comparing VHL-C162F and EV cells indicated 367 downregulated genes and 408 upregulated genes in VHL-C162F cells (Table S2 and Figure 2E). Analysis of VHL-C162F versus WT VHL cells identified 588 downregulated genes and 278 upregulated genes in VHL-C162F cells (Table S3 and Figure 2F). There was an enrichment of hypoxia and EMT pathways by VHL-C162F mutation. Together, these data indicated differences between VHL null (EV) cells and other transfectants, whereas 786-0 cells transfected with WT VHL or VHL-C162F exhibited less variations in their upregulated gene expression profile. Finally, we analyzed the interaction of nine proteins with VHL by STRING database (Figure 2F). These nine proteins including STAT1, EGFR, EPAS1 (or HIF2α), SNAI1, SNAI2 (or Slug), AXL, MAPK1, ZHX2 and USP 13, were selected to represent the different hallmarks enriched in VHL-C162F cells as hypoxia, epithelial mesenchymal transition and proliferation. All these proteins interacting with VHL are in favor of distant metastasis and poor overall survival of patients, angiogenesis, invasion, migration and proliferation [28,29,30].

### 3.3. The High Healing Ability of C162F VHL Mutation Interferes with the Expression of ZHX2

**Figure 3:**
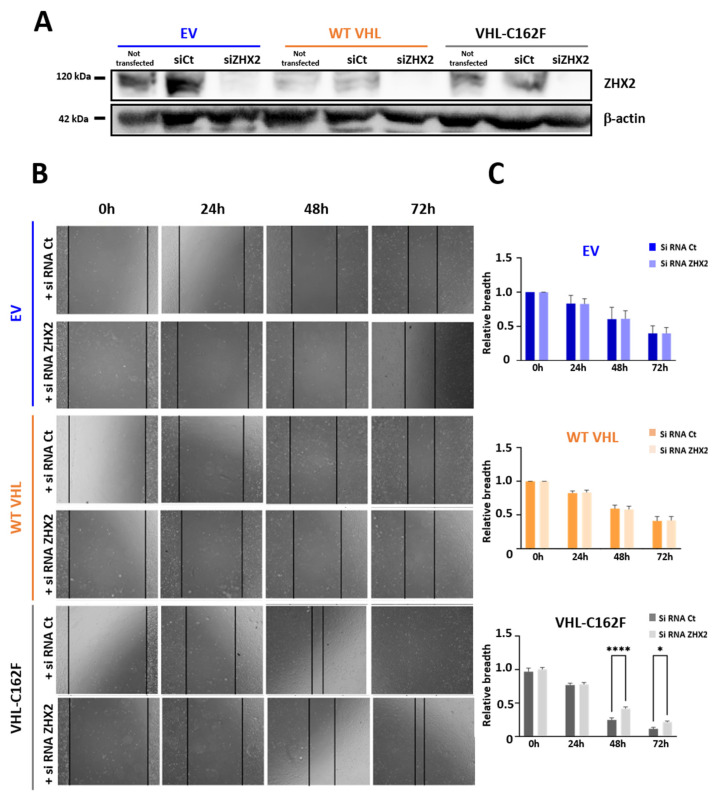
Loss of ZHX2 significantly reduced the healing ability of VHL-C162F mutated cells. (**A**): Western blot analysis of ZHX2 and β actin in cells from wound healing assay. Protein extracts were from EV, WT VHL and VHL-C162F cells not transfected and transfected with siRNA control or siRNA ZHX2. β-Actin is used as a loading control. (**B**): Representative scratch wound images showing the healing ability in EV, WT VHL and VHL-C162F cells transfected with siRNA control or with a mix of siRNA ZHX2. Photographs were taken at 0, 24, 48 and 72 h. (**C**): Graphs represent normalized relative breadth measured at 24, 48 and 72 h for the 3 cells lines transfected with siRNA control or ZHX2 siRNAS (*n* = 3). * *p* ≤ 0.05 and **** *p* ≤ 0.0001 using One Way ANOVA analysis and Bonferroni test. The uncropped bolts are shown in Supplementary Materials Figure S2.

We next focused on ZHX2, a protein of the zinc-fingers and homeoboxes (ZHX) family known to act as an oncogene in RCC in favor of proliferation and migration (Figure 3) [12,13,15].

Using western blot, we confirmed the absence of expression of ZHX2 in cells transfected with the mix of siRNA ZHX2 (Figure 3A and Figure S2). In cells not transfected and in cells transfected with siRNA AllStars negative control (siCt), EV and VHL-C162F cells expressed the most ZHX2 as expected. WT VHL cells not transfected or transfected with siCt had a low expression of ZHX2.

We next asked if ZHX2 interferes with the increased healing ability of VHL-C162F cells using a wound healing assay performed with ZHX2 depleted cells (Figure 3B). We found that VHL-C162F cells transfected with ZHX2 siRNA (siZHX2) showed a significantly lower healing ability than VHL-C162F non-transfected cells at 24, 48 and 72h (Figure 3C).

In absence of the expression of ZHX2, VHL-C162F cells lose their healing ability, whereas WT VHL and EV cells are not affected, suggesting a role of ZHX2 in the healing ability of VHL-C162F cells.

### 3.4. Inhibition of ZHX2 Expression Following Sunitinib Treatment: Impacts on the Healing Ability, Proliferation and Mesenchymal Phenotype

**Figure 4:**
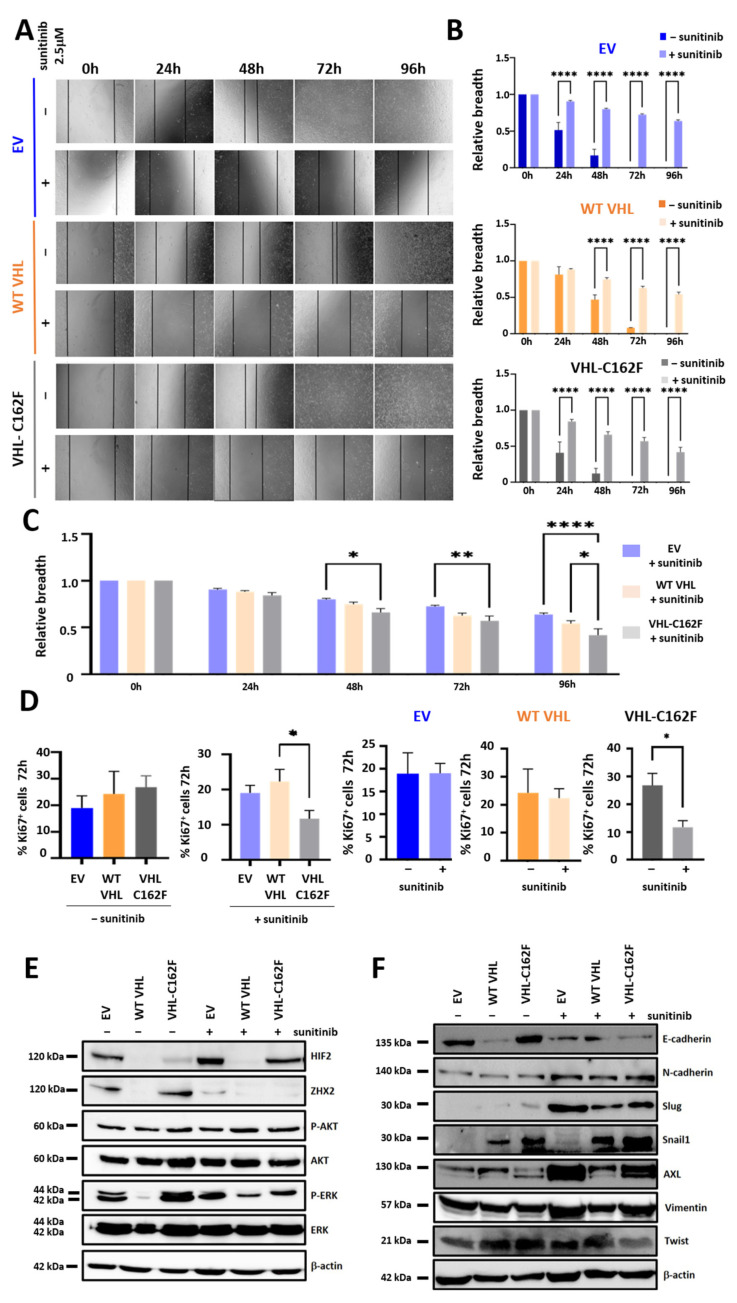
Sunitinib significantly reduced the healing ability of VHL-C162F mutated cells by inhibiting the expression of ZHX2 and inducing a more mesenchymal profile with upregulation of N-cadherin, Slug and AXL. (**A**): Representative scratch wound images showing the reduced healing ability in EV, WT VHL and VHL-C162F cells treated with sunitinib at 2.5 μM. Photographs were taken at 0, 24, 48, 72 and 96 h. (**B**): The healing ability is compared for each cell line not treated versus treated with sunitinib 2.5 μM at 0, 24, 48, 72 and 96 h (*n* = 10). **** *p* ≤ 0.0001 using One Way ANOVA analysis and Bonferroni test. (**C**): The healing ability is compared for the three cell lines treated with sunitinib 2.5 μM (*n* = 10). * *p* ≤ 0.05, ** *p* ≤ 0.01 and **** *p* ≤ 0.0001 using One Way ANOVA analysis and Bonferroni test. (**D**): Intracellular expression of Ki67 in the three cell lines not treated and treated to sunitinib 2.5 μM at 72 h. Graphs represent percentages of Ki67 cells in the three cell lines not treated and treated with sunitinib 2.5 μM at 72 h (*n* = 5). * *p* ≤ 0.05 using One Way ANOVA analysis and Bonferroni test. Graphs represent percentages of Ki67 in EV or WT VHL or C162F cells not treated and treated with sunitinib 2.5 μM at 72 h (*n* = 5). * *p* ≤ 0.05 using Mann-Whitney U test. (**E**): Western blot analysis of HIF2, ZHX2, P-AKT, AKT, P-ERK, ERK and β actin in cells lines not treated and treated with sunitinib 2.5 μM. β-Actin is used as a loading control. Photographs correspond to one representative experiment. Quantifications with ImageJ are in Figure S1. (**F**): Western blot analysis of E-cadherin, N-cadherin, Slug, Snail1, AXL, Vimentin, Twist and β actin in cells lines not treated and treated with sunitinib 2.5 μM. β-Actin is used as a loading control. Photographs correspond to one representative experiment. Quantifications with ImageJ are in Figure S3. The uncropped bolts are shown in Supplementary Materials Figures S4–S9.

As sunitinib treatment is known to inhibit the proliferation of several cancer cell lines [31,32], we asked whether sunitinib can impact the healing ability of VHL-C162F cells by a scratch test of wound healing assay at high confluence (Figure 4A) at 24, 48, 72 and 96 h. For all cell lines independently of the VHL status, the healing ability was significantly slowed down with sunitinib treatment (Figure 4A,B).

We then compared the healing ability between the three cell lines treated with sunitinib at 0, 24, 48, 72 and 96 h. Data depicted in Figure 4C showed that all cell lines are impacted by sunitinib treatment. Healing ability is significantly slowed down regardless of VHL status. The healing ability is similar for EV cells and C162F cells treated with sunitinib. It slows down from 24 h whereas for WT VHL it slows down from 48 h. We next compared the ability of proliferation by measuring Ki67^+^ cells for the 3 cell lines after treatment with sunitinib at 72 h (Figure 4D). No significant difference of proliferation was observed between the 3 untreated cell lines at 72 h. After 72 h of treatment with sunitinib at 2.5 μM, proliferation was reduced in VHL-C162F cells with a significant difference versus WT VHL cells and also versus untreated VHL-C162F. In contrast, no difference was observed for WT VHL cells following sunitinib treatment versus untreated WT VHL cells. There is also no significant difference between EV cells treated with sunitinib versus untreated EV cells. Western blot analysis data (Figure 4E and Figures S3–S6) indicated a significant downregulation of expression of ZHX2 while no change was observed in the expression of P-AKT for the 3 cells lines after 15 days of treatment with sunitinib. P-ERK was downregulated in VHL-C162F cells with sunitinib treatment, whereas upregulation of P-ERK in WT VHL cells with sunitinib treatment in favor of proliferation was observed (Figure 4E, Figures S3 and S6). These results correlated with the percentage of Ki67^+^ cells detected in WT VHL cells treated with sunitinib (Figure 4D). Finally, western blot analysis was in favor of a more mesenchymal profile (Figure 4F and Figures S7–S9) as illustrating a downregulation of E cadherin in treated VHL-C162F cells associated with upregulation of N-cadherin, Slug and AXL (Figure 4F, Figures S3, S7 and S8). Expression of Vimentin and Twist were not significantly changed by the sunitinib treatment for the three cells lines (Figure 4F, Figures S3 and S9).

In conclusion, sunitinib treatment slows down proliferation of VHL-C162F cells through inhibition of ZHX2 and P-ERK and induced a decrease in healing ability. It also shapes the profile of VHL-C162F cells in a more mesenchymal phenotype.

## 4. Discussion

It is well established that *VHL* mutations contribute to tumorigenesis and influence the ccRCC development in VHL disease [33]. Multiple studies showed that the VHL pathway targets also ZHX2 [6,13,15].

Here, using a human VHL mutated RCC cell line expressing the VHL-C162F mutation on a doxycycline-inducible system, we demonstrated that this mutation induces a pseudo-hypoxic state associated with a high expression of ZHX2 in favor of proliferation, migration and invasion. The VHL-C162F mutation has no impact on morphology of cells. Nevertheless, the mutation C162F induces a less significant pronounced potential to grow as a single cell and as colonies compared to the WT-VHL-and EV transfected 786-0 cells. The VHL-C162F cells have a similar ability to migrate as WT VHL cells in transwell experiments performed at low density.

Our data indicate that the mutation C162F confers a significant higher healing ability by comparison with WT VHL and EV cells. It may impact the behavior of the VHL-C162F cells to spread and migrate. It has been already published that cell density is an important factor to consider when comparing the VHL status of the cells [34,35]. At low density, VHL-C162F cells seems to be less aggressive than at high density.

To further elucidate the mechanism by which this mutation interferes with gene transcription, we performed a global transcriptional analysis. We demonstrated that transfection by WT VHL and VHL-C162F mutation induced transcriptional changes in VHL-C162F cells. We demonstrated an enrichment of genes regulated by hypoxia as well as genes implicating epithelial and mesenchymal transition (EMT) in the VHL-C162F cells. This is reinforced by the String analysis protein interaction showing interaction between VHL and the nine main proteins implicated in hypoxia and EMT.

As ZHX2 is known to be implicated in cell proliferation, migration and invasion in ccRCCs, we investigated the possible involvement of ZHX2 to explain the higher healing ability in VHL-C162F cells. We did a knocking down of ZHX2 by transfection with a mix of siRNA. In absence of expression of ZHX2, VHL-C162F cells significantly lose their healing ability. Moreover, healing ability in WT VHL cells and in EV cells were not significantly impacted by the knocking down of ZHX2. This is consistent with a role of ZHX2 in the healing ability associated with the VHL-C162F mutation. The mechanism of how VHL-C162F increases ZHX2 expression is not well established, although earlier, a genome-wide screen identified ZHX2 as a hydroxylation-dependent VHL substrate that promotes NF-κB activity and ccRCC tumorigenesis [13] The same authors have demonstrated that ZHX2 is regulated by VHL through prolyl hydroxylation and proteasomal degradation.

We then analyzed the in vitro impact of the sunitinib treatment on cells with VHL-C162F mutation by measuring the healing ability. We showed that sunitinib is efficient to slow down healing ability in VHL-C162F cells but also in WT VHL and EV cells regardless of VHL status. Using western blot, we showed downregulation of ZHX2 in sunitinib treated cells. Sunitinib may potentially slow down healing and proliferation in VHL-C162F cells by inhibiting P-ERK. The KI67+ cells proliferation test indicates that VHL-C162F cells requires to be treated at least 72h with sunitinib to get a slowed down proliferation. This slowed down proliferation is in accordance with the observation that in several clinical trials, a clinical beneficial response to TKI treatment has been observed. However, patients can develop resistance to TKI therapy by mechanisms which remain largely unknown [36].

Moreover, sunitinib confers a more mesenchymal phenotype in VHL-C162F cells in favor of disease progression. In our model, sunitinib significantly downregulates the epithelial marker E-cadherin and upregulates the three mesenchymal markers N-cadherin, Slug and AXL in VHL-C162F cells. No change of expression of Vimentin, Twist and Snail1 was observed in VHL-C162F cells. Similar results were obtained in a model of colorectal cancer where sunitinib induces EMT with downregulation of E-cadherin and upregulation of Slug and Zeb1 in favor of tumoral progression [37]. It was also reported in 786-0 cells (VHL−/−) treated with sunitinib, an induction of EMT markers via activation of EGFR [38]. Decreased expression of E-cadherin by immunohistochemistry staining was already described in sunitinib resistant tumors [39]. The survival period is significantly longer in patients whose tumors do not express Slug [40]. Recently, we have demonstrated an upregulation of AXL and PDL1 that was associated with a significant low survival for RCC patients [29]. The downregulation of E-cadherin leads to alterations in cell adhesions and confers them with an invasive mesenchymal phenotype. In ccRCCs, SNAIL1 is known to induce EMT by repressing E-cadherin gene expression and enhancing N-cadherin expression [41,42,43,44]. Therefore, based on our model, it is tempting to speculate that the induction of EMT markers such as N cadherin, Slug and AXL in VHL-C162F cells with sunitinib treatment may be associated with RCC and a lower survival rate for VHL-C162F patients. These observations have to be considered to design the best treatment for patients with VHL-C162F disease.

## 5. Conclusions

We have previously provided evidence that a discrete VHL point mutation, the most common *VHL* point mutation R167Q in hereditary VHL disease, interferes with tumor plasticity and may impact cell behavior by exacerbating phenotypic switching [11]. In the current studies, we investigated the role of VHL-C162F known to be one of the most severe phenotypes for patients with VHL disease.

Most *VHL* mutations in VHL disease affect the oxygen dependent regulation of HIF1/2α, resulting in the accumulation of HIF1/2α as well as ZHX2, a target of VHL driving ccRCCs. On the basis of the results of wound healing assays, VHL-C162F cells showed a higher healing ability than WT-VHL and EV cells, most likely due to high expression of ZHX2. Global gene expression profiling demonstrates an enrichment of hypoxia and EMT hallmarks in VHL-C162F cells as compared with WT VHL cells. More importantly, in VHL-C162F cells, sunitinib treatment significantly downregulates proliferation and healing ability via the downregulation of ZHX2 and P-ERK, but confers a more mesenchymal phenotype resulting in downregulation of E-cadherin and upregulation of mesenchymal markers including N Cadherin, Slug and AXL. The increased migration observed in VHL-C162F could be in fact due to the acquisition of the mesenchymal phenotype. Epithelial-to-mesenchymal transition (EMT) is known to play crucial roles in metastatic dissemination. Therefore, the VHL-C162F-induced upregulation of EMT markers may presumably help the cells to gain migratory and invasive traits.

In this context, sunitinib therapy may promote disease progression in VHL-C162F patient.

The results of the current studies may guide the development of new therapy targeting ZHX2 but also mesenchymal markers of EMT to improve patient’s chances of survival with VHL-C162F mutation.

## Data Availability

RNA Seq data presented in this study are available on request from the corresponding authors.

## Supplementary Materials

The following supporting information can be downloaded at: https://www.mdpi.com/article/10.3390/cancers16010034/s1, Table S1: List of primary antibodies used in western blots. Table S2: List of differentially expressed genes EV vs. VHL-C162F. Table S3: List of differentially expressed genes WT VHL vs VHL-C162F. Figure S1: Uncropped Western Blot of Figure 1B: HIF2, VHL and β-actin. Figure S2: Uncropped Western Blot of Figure 3C: ZHX2 and β-actin. Figure S3: Quantifications of western blots analysis of expression of ZHX2, phospho-AKT and AKT, phospho-ERK and ERK of Figure 4E, E-cadherin, N-cadherin, Slug, Snail1, AXL, Vimentin and Twist of Figure 4F. Figure S4: Uncropped Western Blot of Figure 4E: HIF2 and ZHX2. Figure S5: Uncropped Western Blot of Figure 4E: P-AKT, AKT and β-actin. Figure S6: Uncropped Western Blot of Figure 4E: P-ERK and ERK. Figure S7: Uncropped Western Blot of Figure 4F: E-cadherin, N-cadherin and β-actin. Figure S8: Uncropped Western Blot of Figure 4F: Slug, Snail1 and AXL. Figure S9: Uncropped Western Blot of Figure 4F: Vimentin and Twist.
