# Supplementary material for: Sunitinib Treatment of VHL C162F Cells Slows Down Proliferation and Healing Ability via Downregulation of ZHX2 and Confers a Mesenchymal Phenotype
Source: Cancers (Basel). 2023 Dec 20;16(1):34. doi: 10.3390/cancers16010034 (PMC10778532; doi:10.3390/cancers16010034)
Supplement: Supplementary file 1 [file cancers-16-00034-s001.zip › cancers-2683197-supplementary final.pdf]

Supplementary Materials: Sunitinib Treatment of VHL C162F Cells Slows Down Proliferation and Healing Ability via Downregulation of ZHX2 and Confers a Mesenchymal Phenotype

Stéphanie Buart, M'boyba Khadija Diop, Isabelle Damei and Salem Chouaib

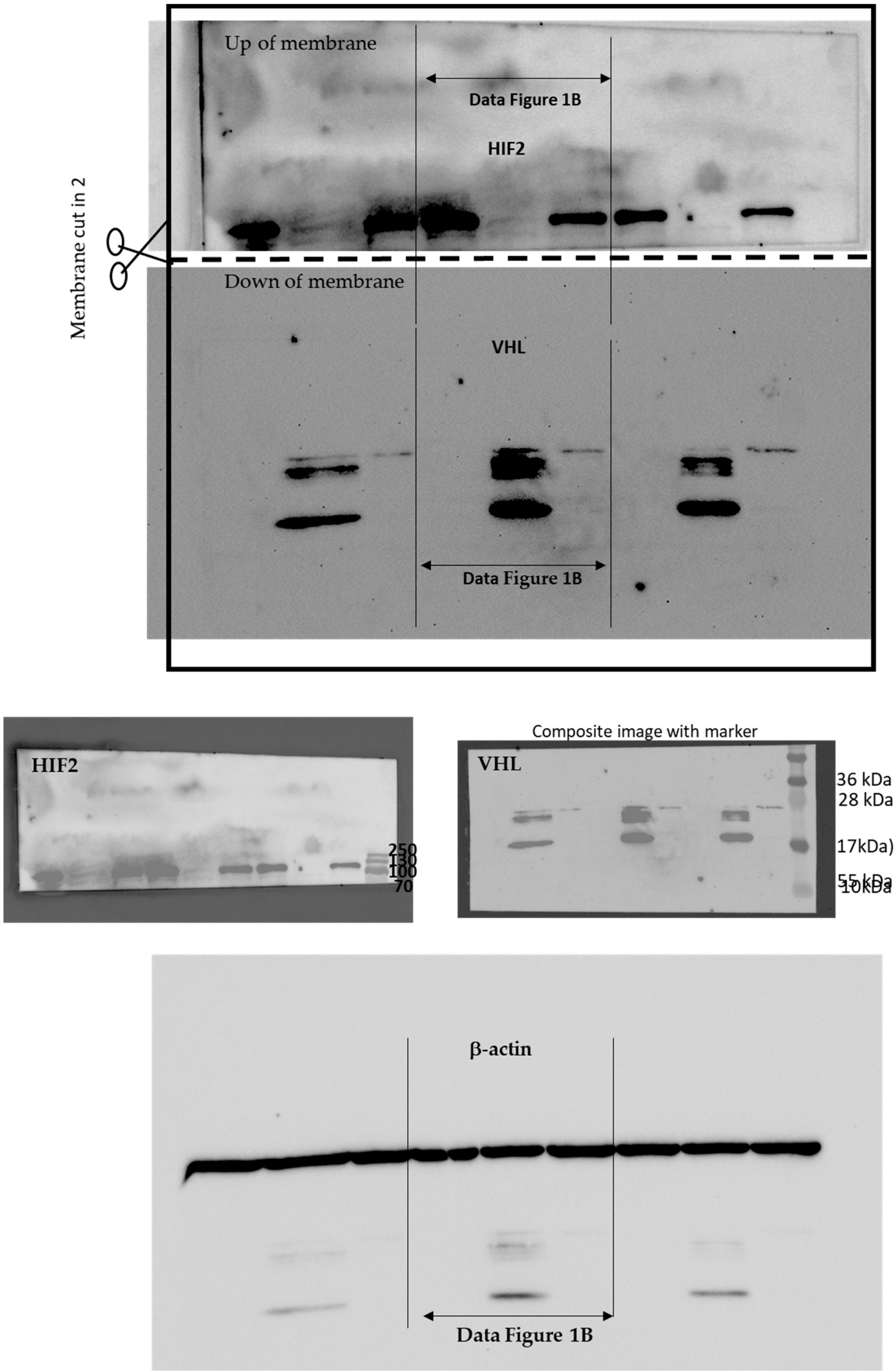

Figure S1. Uncropped Western Blot of Figure 1B: HIF2, VHL and β-actin.

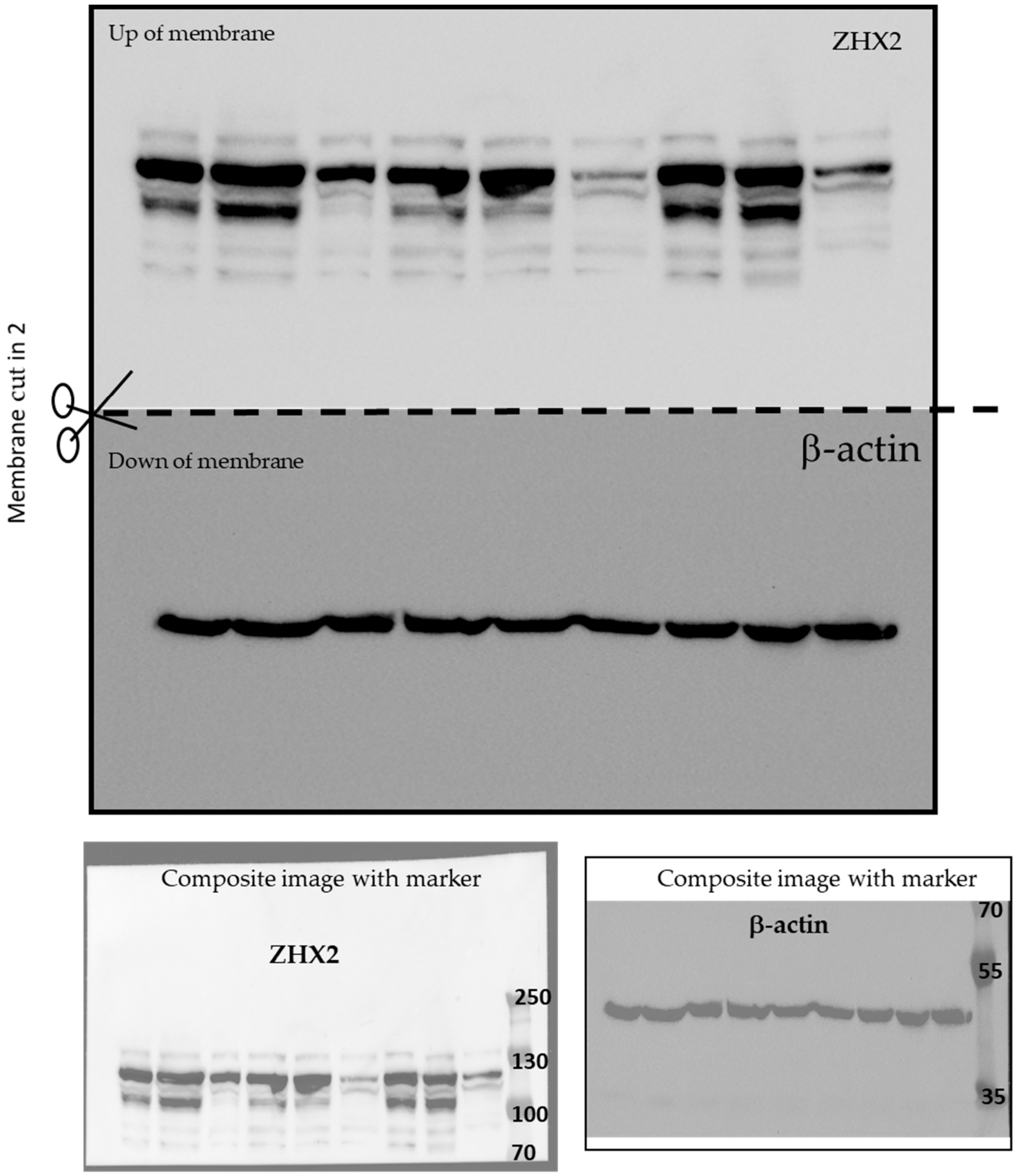

Figure S2. Uncropped Western Blot of Figure 3C: ZHX2 and β-actin.

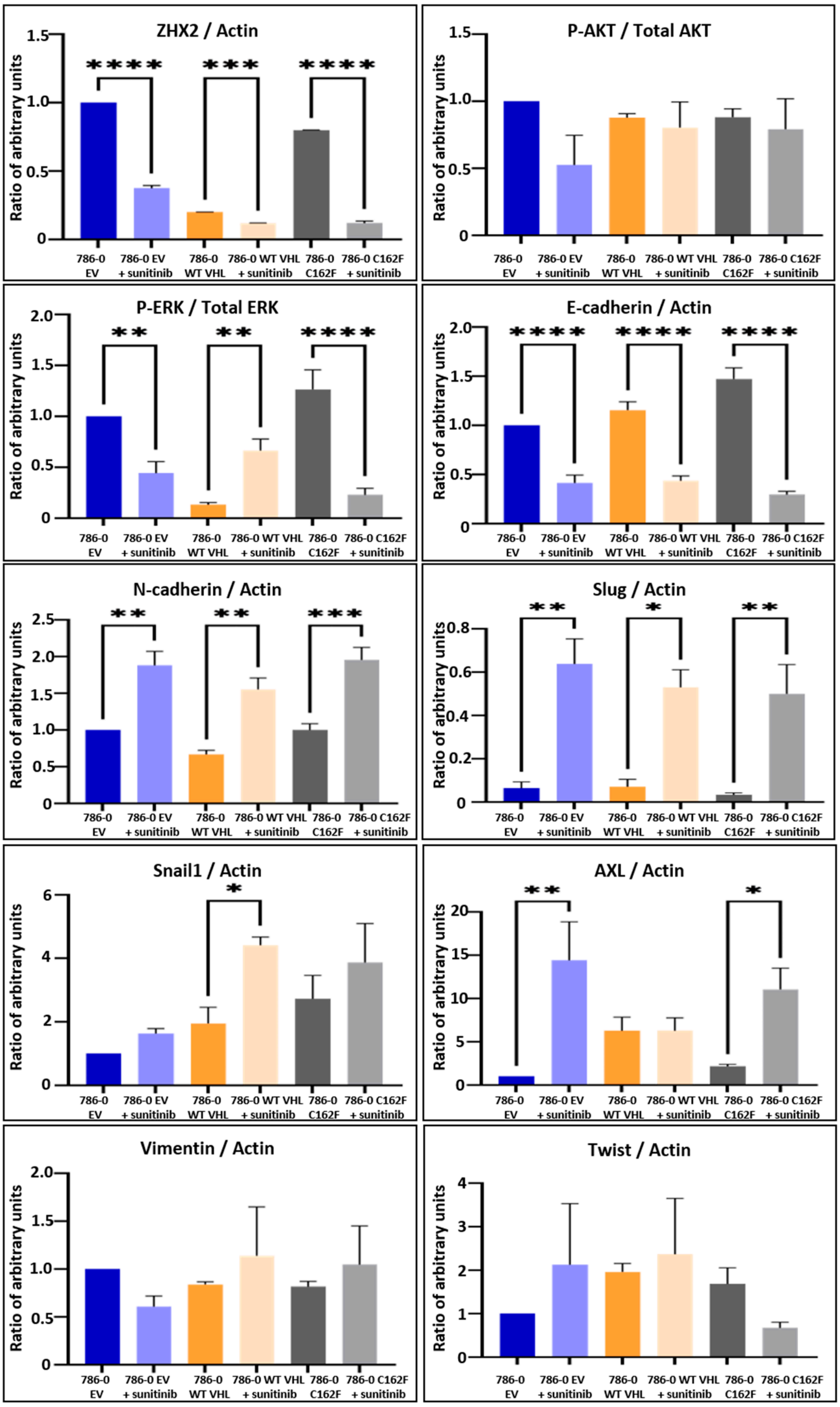

Figure S3. Quantification of western blots analysis of expression of ZHX2, phospho-AKT and AKT, phospho-ERK of Figure 4E and ERK, E-cadherin, N-cadherin, Slug, Snail1, AXL, Vimentin and Twist of Figure 4F. Quantification of band intensities was performed using ImageJ. Graphs represent the mean ± sem normalized with 786-0 EV not treated with sunitinib except for Slug/Actin western blot analysis not normalized (n = 3 for each representation except for Twist n = 4). *, $p \leq 0.05$, **, $p \leq 0.01$, ***, $p \leq 0.001$ and****, $p \leq 0.0001$ using one way ANOVA and Bonferroni test.

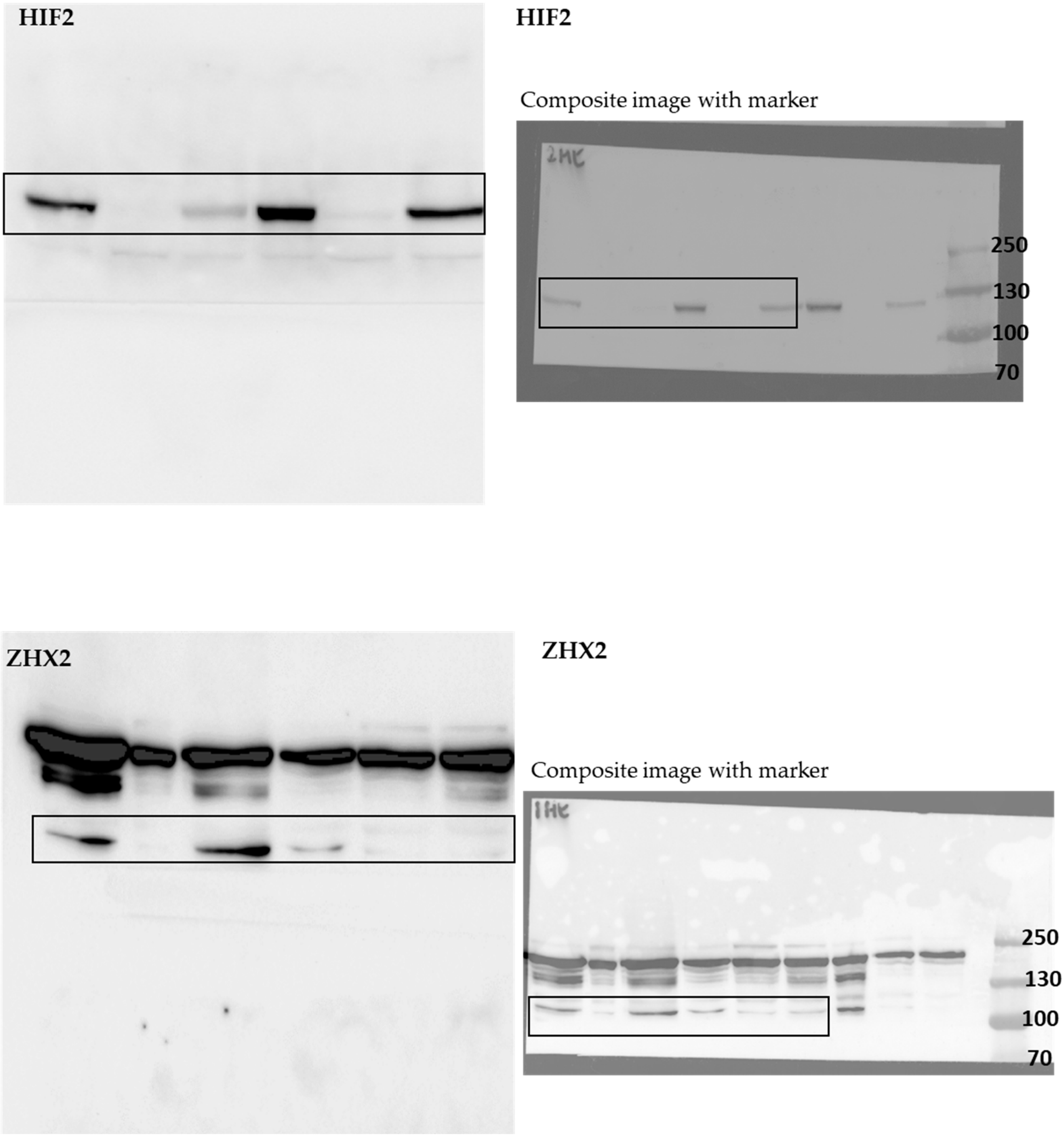

Figure S4. Uncropped Western Blot of Figure 4E: HIF2 and ZHX2.

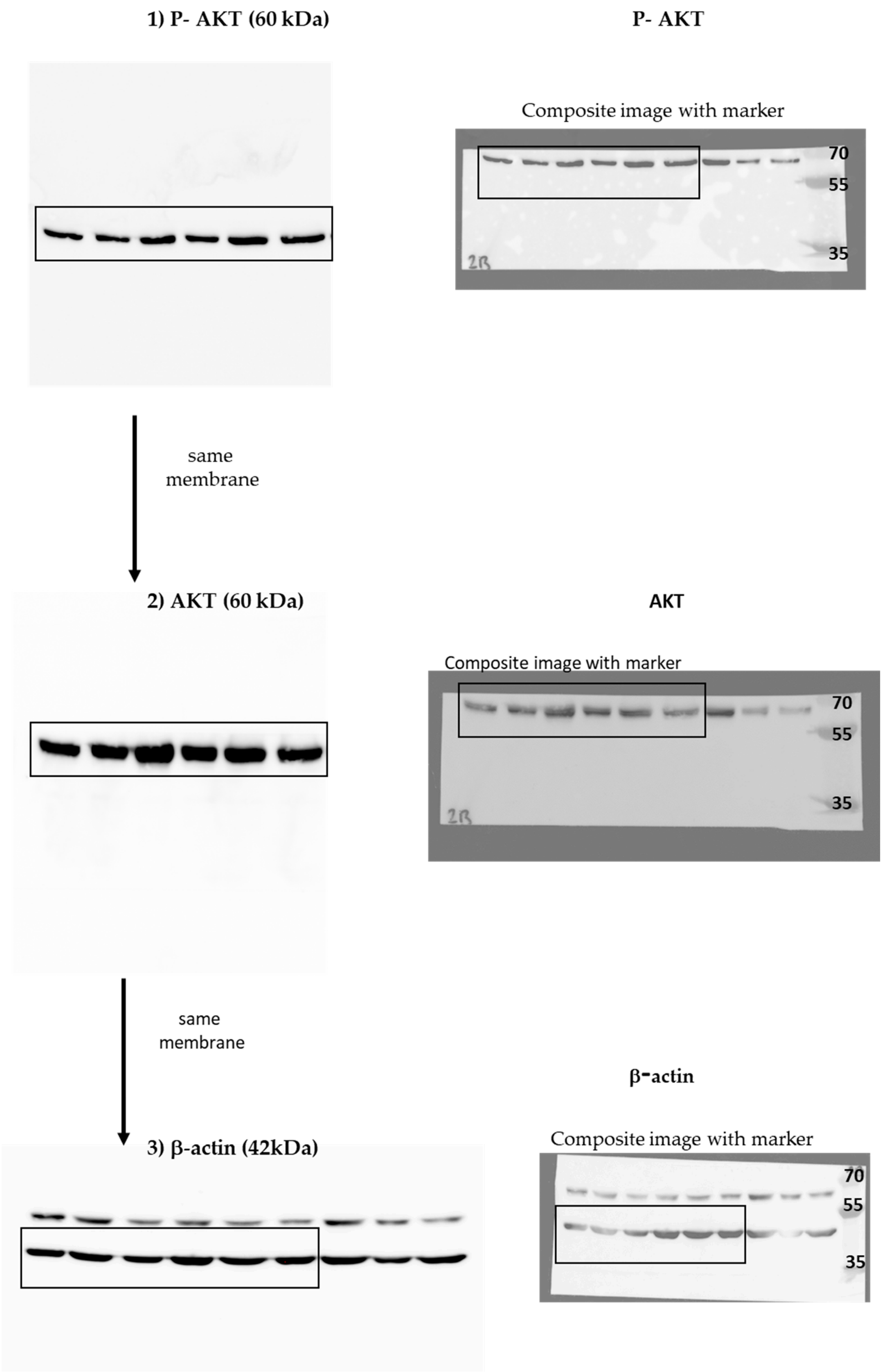

Figure S5. Uncropped Western Blot of Figure 4E: P-AKT/AKT and β-actin.

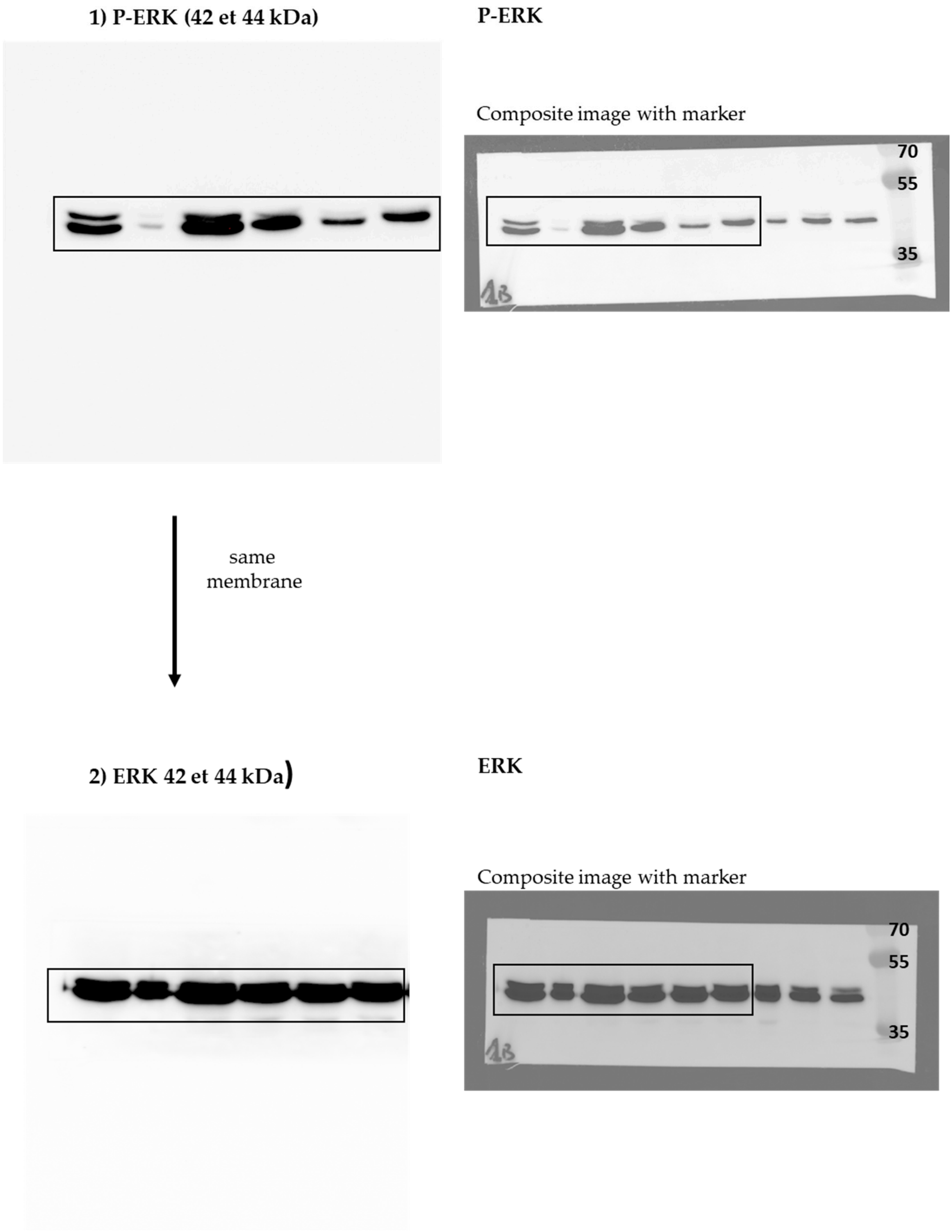

Figure S6. Uncropped Western Blot of Figure 4E: P-ERK and ERK.

E-cadherin (135 kDa)

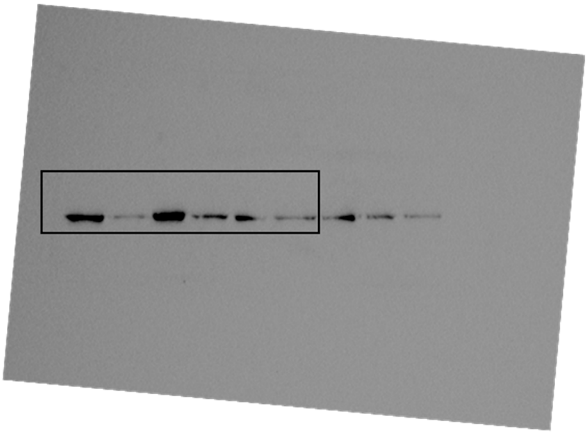

Composite image with marker

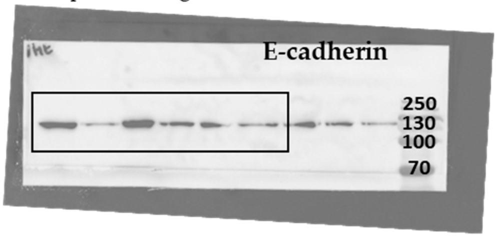

N-cadherin (140 kDa)

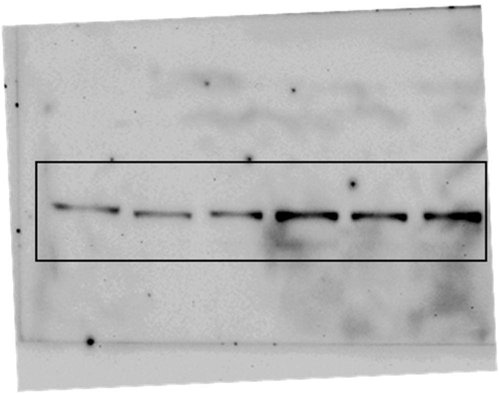

Composite image with marker

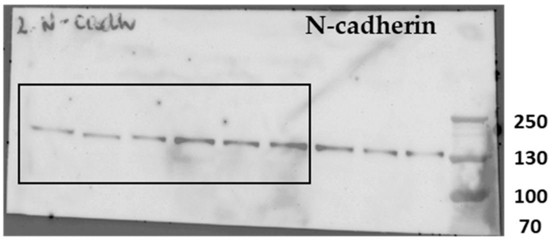

β-actin (42 kDa)

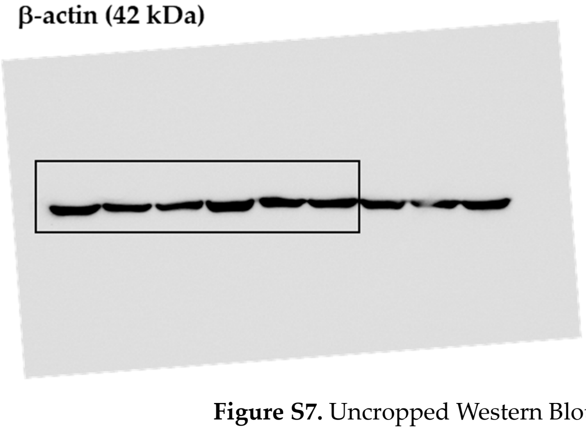

Composite image with marker **β-actin**

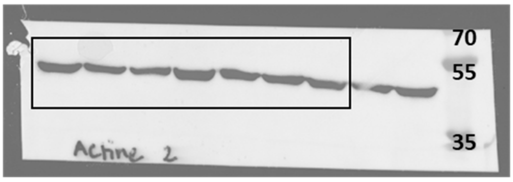

Figure S7. Uncropped Western Blot of Figure 4F: E-cadherin, N-cadherin and β-actin.

Slug (30 kDa)

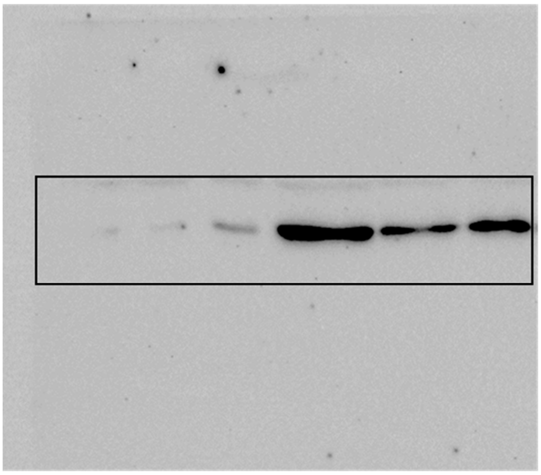

Slug

Composite image with marker

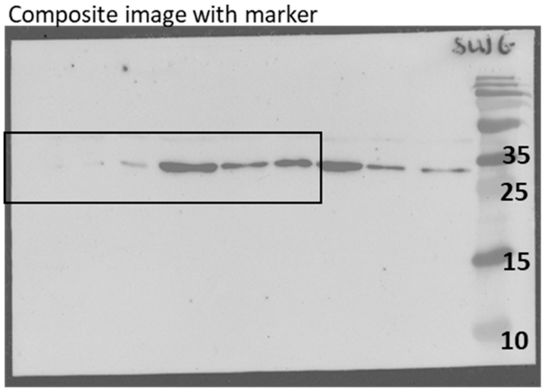

Snail1 (30 kDa)

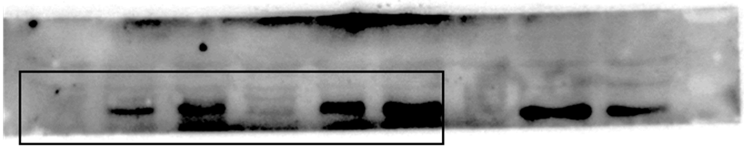

Snail1

Composite image with marker

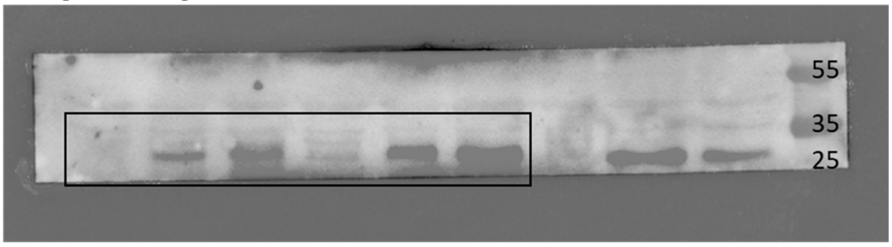

AXL (130 kDa)

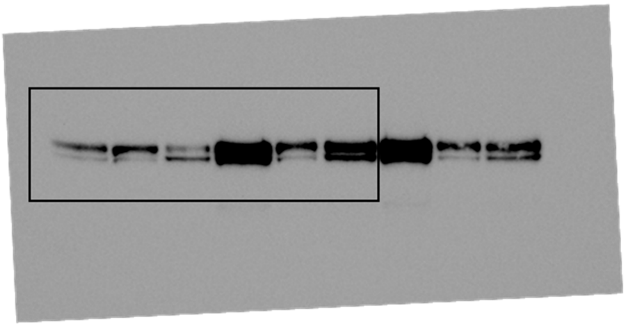

AXL

Composite image with marker

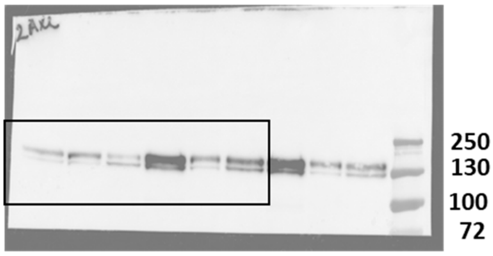

Figure S8. Uncropped Western Blot of Figure 4F: Slug, Snail1 and AXL.

Vimentin (57 kDa)

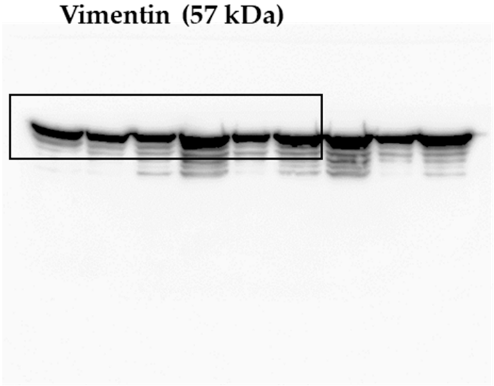

Vimentin

Composite image with marker

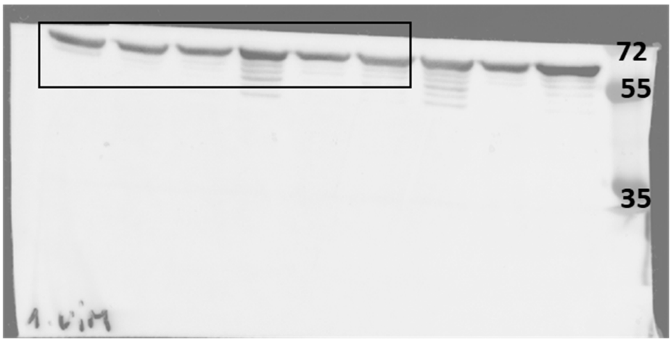

Twist (21 kDa)

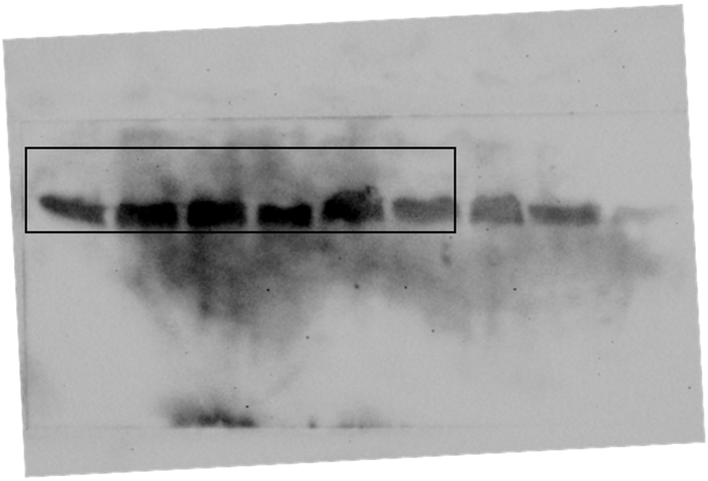

Twist

Composite image with marker

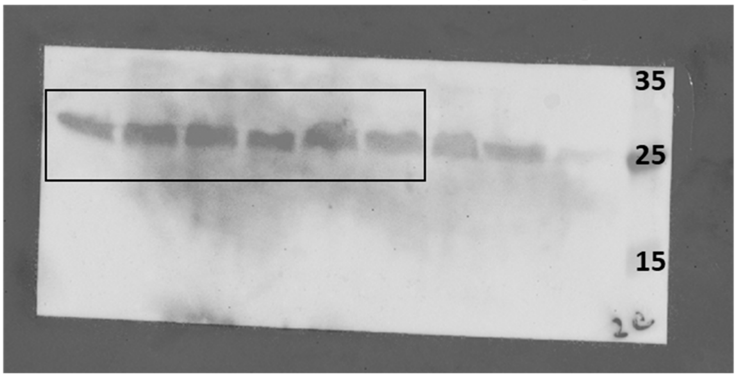

Figure S9. Uncropped Western Blot of Figure 4F: Vimentin and Twist.

Table S1. List of primary antibodies used in western-blot analysis.

Anti VHL	Rabbit Ab	2738	Cell Signaling Technology; Danvers, MA, USA
Anti HIF2a	Rabbit Ab	7096S	Cell Signaling Technology; Danvers, MA, USA
Anti ZHX2	Rabbit Ab	GTX112232	GeneTex, San Antonio, TX, USA
Anti P-AKT (Ser473)	Rabbit Ab	4060	Cell Signaling Technology; Danvers, MA, USA
Anti AKT	Mouse Ab	9272	Cell Signaling Technology; Danvers, MA, USA
Anti P-ERK	Rabbit Ab	9102	Cell Signaling Technology; Danvers, MA, USA
Anti ERK1/2	Mouse Ab	4696	Cell Signaling Technology; Danvers, MA, USA
Anti E-cadherin	Rabbit Ab	3195S	Cell Signaling Technology; Danvers, MA, USA
Anti N-cadherin	Rabbit Ab	4061	Cell Signaling Technology; Danvers, MA, USA
Anti Slug	Rabbit Ab	9585 clone C19G7	Cell Signaling Technology; Danvers, MA, USA
Anti AXL	Rabbit Ab	8661S	Cell Signaling Technology; Danvers, MA, USA
Anti Snail1	Rabbit Ab	3895	Cell Signaling Technology; Danvers, MA, USA
Anti Vimentin	Mouse Ab	SC6260	Santa Cruz Biotechnology ; Dallas, TX, USA
Anti Twist	Mouse Ab	ab50887	Abcam Cambridge, UK
Anti b-actin-HRP	Mouse Ab	A3854 clone AC-15	Sigma-Aldrich, St. Louis, MO, USA

Table S2. List of differentially expressed genes EV vs VHL-C162F.

Table S3. List of differentially expressed genes WT VHL vs VHL-C162F.

Tables S2–S3. are provided separately, attached as Excel files.
